# Extraction Condition Optimization, Quantitative Analysis, and Anti-AD Bioactivity Evaluation of Acorn Polyphenols from *Quercus variabilis, Quercus aliena*, and *Quercus dentata*

**DOI:** 10.3390/ijms251910536

**Published:** 2024-09-30

**Authors:** Jianing Du, Zhengkun Han, Longyi Ran, Taiyu Zhang, Junru Li, Huiying Li

**Affiliations:** Beijing Key Laboratory of Food Processing and Safety in Forestry, College of Biological Sciences and Technology, Beijing Forestry University, Beijing 100083, China; djn15661258236@bjfu.edu.cn (J.D.); zhengkunh1005@126.com (Z.H.); rly2229@bjfu.edu.cn (L.R.); zhangtaiyu1120@163.com (T.Z.); 18395270921@163.com (J.L.)

**Keywords:** acorn polyphenol, endogenous phenolics, LC-MC/MS, quercetin, Alzheimer’s disease

## Abstract

In the present study, *Quercus variabilis (Q. variabilis), Quercus aliena (Q. aliena),* and *Quercus dentata (Q. dentata)* acorn kernels were taken as the research objects, and the free polyphenols and bound polyphenols in acorn kernels were extracted using improved ultrasound-assisted ethanolic and alkaline extraction methods, after which the contents of gallic acid, quercetin, azelaic acid, ellagic acid, and ferulic acid were quantified by LC-MC/MS. The results demonstrated that *Q. variabilis* and *Q. aliena* acorns were suitable as raw materials to extract ellagic acid, the contents of ferulic acid and bound gallic acid in them were different, and *Q. aliena* acorns were more suitable for the research of gallic acid, but not for azelaic acid. Results on APP/PS1 transgenic mice verified that five polyphenols significantly suppressed the progression of AD. This study provides a theoretical basis for the drug development of acorn polyphenols.

## 1. Introduction

Acorns are rich in polyphenolic substances, mainly composed of gallic acid derivatives, and also contain a significant amount of γ-tocopherol [1]. The polyphenolic composition in acorns is influenced by the geographical location, chemical characteristics, and biological characteristics of the oak trees. Tannins are among the main compounds in acorns, but analysis reveals that in *Quercus* (*Q.*) robur and *Q. petraea* acorns, tannins are the predominant polyphenolic components, whereas in *Q. pubescens* acorns, the content of non-tannin phenols is relatively higher. Additionally, in *Q. rubra* acorns, the tannin content is slightly lower than in *Q. robur* and *Q. petraea*, while the levels of other phenolic compounds are higher [2]. Over 30 phenolic compounds have been identified in different acorn extracts, including gallic acid derivatives, tannic acid derivatives, tannins, catechins, quercetin, ajulemic acid, and ferulic acid [3].

Acorn polyphenols exhibit various biological activities, including antioxidant, anti-tumor, anti-inflammatory, lipid-lowering, hypoglycemic, and antibacterial effects [4]. These polyphenols contain functional components such as ellagic acid, tannic acid, and flavonoids, possessing potent antioxidant properties that scavenge free radicals and protect cells from oxidative damage [5]. Notably, acorn polyphenols significantly inhibit the expression levels of acetylcholinesterase (AChE) and butyrylcholinesterase (BChE) in human neuroblastoma cells (SH-SY5Y), indicating their neuroprotective potential for alleviating symptoms associated with Alzheimer’s disease and other neurodegenerative diseases [6]. Furthermore, their abilities to modulate inflammatory responses and glucose metabolism indicate a broader therapeutic scope, potentially addressing AD and related metabolic disorders [3,7,8,9,10].

Alzheimer’s disease (AD) represents one of the most prevalent neurodegenerative disorders worldwide, characterized by progressive cognitive decline and memory loss. The etiology of AD is complex, involving multiple factors such as amyloid-beta (Aβ) plaque accumulation, oxidative stress, and inflammation [11,12]. One significant consequence of Aβ accumulation is oxidative stress, marked by an imbalance between the production of reactive oxygen species (ROS) and the brain’s antioxidant defenses [13]. Oxidative stress in AD contributes to neuronal damage and is closely linked to the formation and propagation of Aβ plaques [14]. The brain’s high oxygen consumption rate, along with its abundant lipid content and relatively weaker antioxidant defenses, makes it particularly susceptible to oxidative damage, which exacerbates AD pathology [15]. Furthermore, the interaction between Aβ plaques and microglial cells, the brain’s resident immune cells, triggers an inflammatory response, leading to the release of pro-inflammatory cytokines and further neuronal damage [16]. This inflammatory process is believed to amplify the neurodegenerative effects of Aβ, creating a vicious cycle that accelerates the progression of AD [17].

Acorn polyphenols possess various biological activities and have broad application prospects. However, research on the polyphenol composition of commonly found *Quercus* species in China is limited. In this study, we utilized physicochemical techniques to crush and identify the acorns of three common species in China, *Q. variabilis*, *Q. aliena*, and *Q. dentata*. We conducted representative polyphenol compound content detection and analysis. Then, APP/PS1 transgenic mice model experiments were conducted to explore the effect of acorn polyphenols on Alzheimer’s disease and to provide a reference for the development of acorn polyphenol resources and drug development in Alzheimer’s disease.

## 2. Results

### 2.1. Determination of Polyphenols in Main Acorns

According to the methods and conditions described in the Section 4.3, seven levels of gallic acid, ellagic acid, ferulic acid, azelaic acid, and quercetin were determined, and their responsivity and corresponding sample concentration data are shown in Supplementary Table S1. Linear regression was performed on the obtained data, and the standard curve was drawn, as shown in Supplementary Table S1. The experimental results showed that the absolute coefficients were all R^2^ > 0.9995, indicating that the five standards had good linearity within the determination range. Under certain chromatographic conditions, any substance has a certain retention time and can be used for the identification of sample components. Therefore, in order to identify each sample, the retention time of each standard under different concentrations was recorded and the average retention time of the sample was calculated during the above experiment, as shown in Supplementary Table S2.

**Figure 1:**
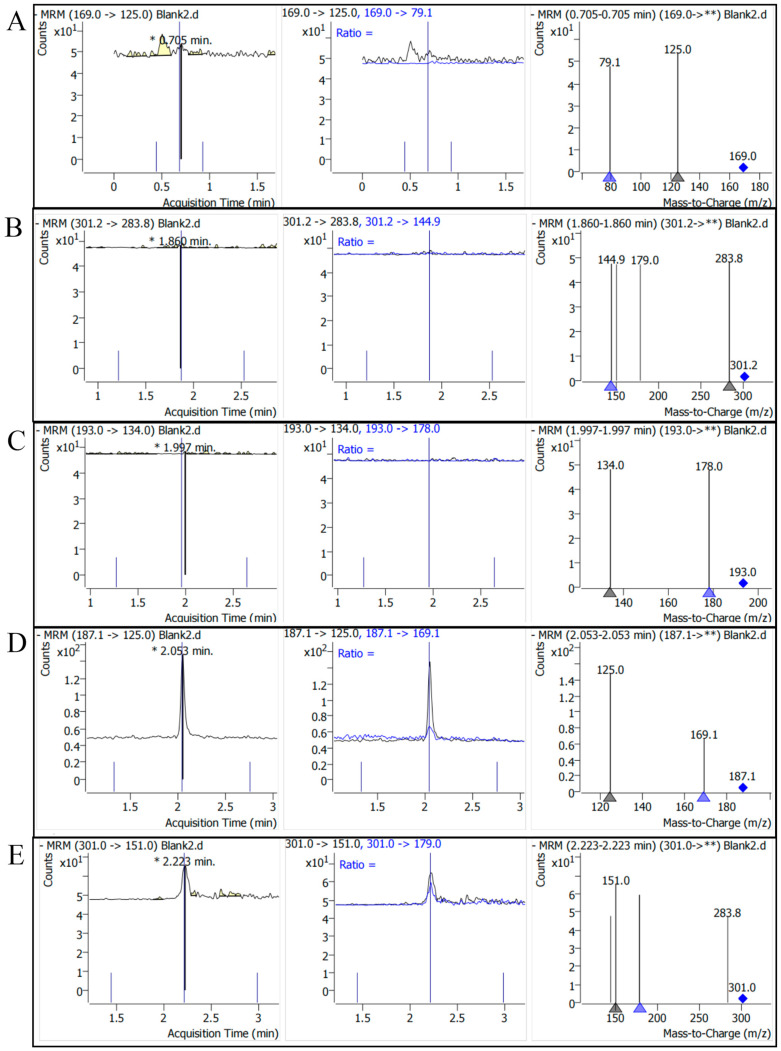
Mass spectra of major polyphenols in acorn kernels. (**A**) Detection of gallic acid; (**B**) detection of ellagic acid; (**C**) detection of ferulic acid; (**D**) detection of azelaic acid; (**E**) detection of quercetin. Asterisk (*): Indicates a significant peak at a specific retention time, representing the characteristic of the target compound at that time point. Double asterisks (**): Represent a full scan of all fragment ions from the parent ion. Triangle (▲): Marks specific fragment ions. Diamond: Marks the parent ion (the original mass-to-charge ratio of the target compound).

According to the above method, each concentration gradient of the standard sample is detected, and the characteristic fragment ion mass-to-charge ratio of each standard can be obtained (Figure 1), as shown in Supplementary Table S3.

### 2.2. Results of Liquid Chromatography

**Figure 2:**
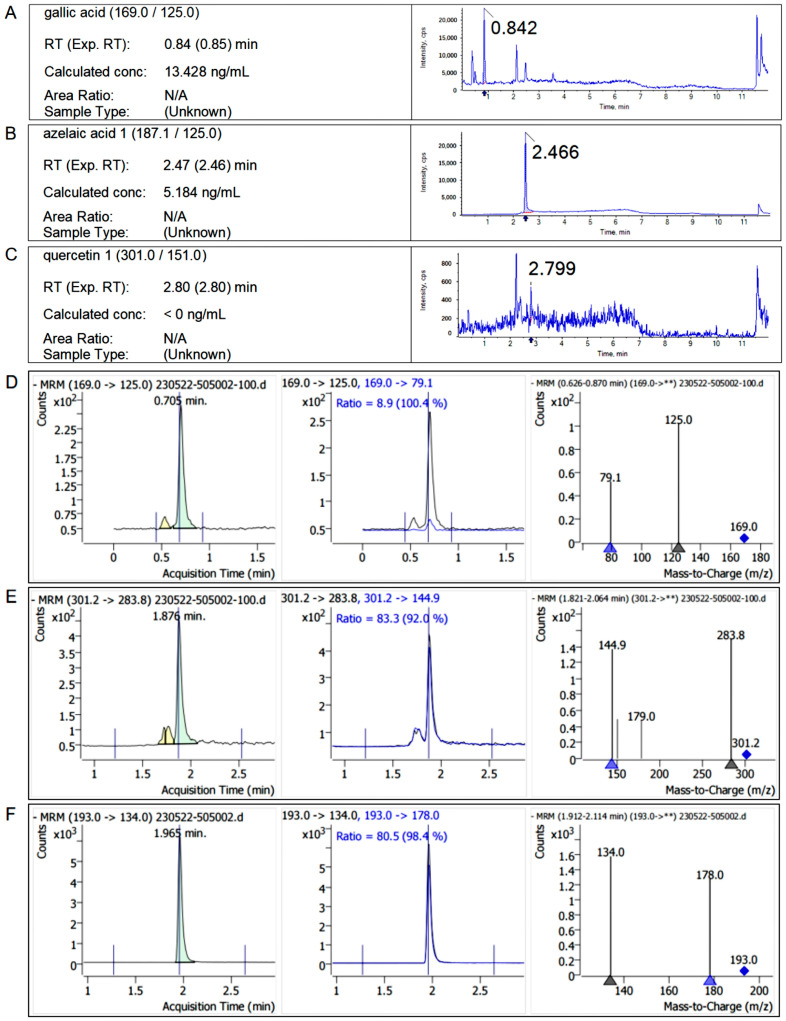
Chromatograms of free polyphenols and bound polyphenols in *Quercus variabilis* kernel. (**A**–**C**) The chromatograms of free gallic acid, azelaic acid, and quercetin in the kernel extract of *Quercus variabilis*. (**D**–**F**) The chromatograms of bound gallic acid, ellagic acid, and ferulic acid in the kernel extract of *Quercus variabilis*. Double asterisks (**): Represent a full scan of all fragment ions from the parent ion. Triangle (▲): Marks specific fragment ions. Diamond: Marks the parent ion (the original mass-to-charge ratio of the target compound).

**Figure 3:**
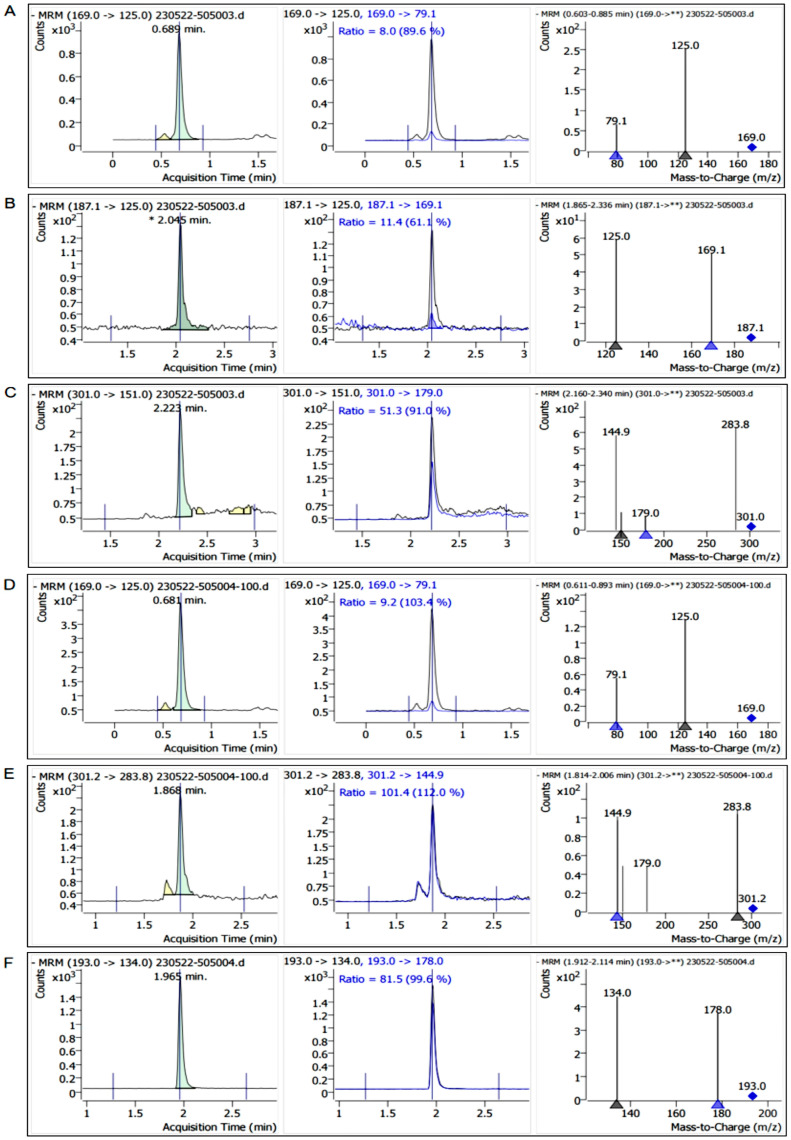
Chromatograms of free polyphenols and bound polyphenols in *Quercus aliena* kernel. (**A**–**C**): The chromatograms of free gallic acid, azelaic acid, and quercetin in the kernel extract of *Quercus aliena*. (**D**–**F**): The chromatograms of bound gallic acid, ellagic acid, and ferulic acid in the kernel extract of *Quercus aliena*. Asterisk (*): Indicates a significant peak at a specific retention time, representing the characteristic of the target compound at that time point. Double asterisks (**): Represent a full scan of all fragment ions from the parent ion. Triangle (▲): Marks specific fragment ions. Diamond: Marks the parent ion (the original mass-to-charge ratio of the target compound).

**Figure 4:**
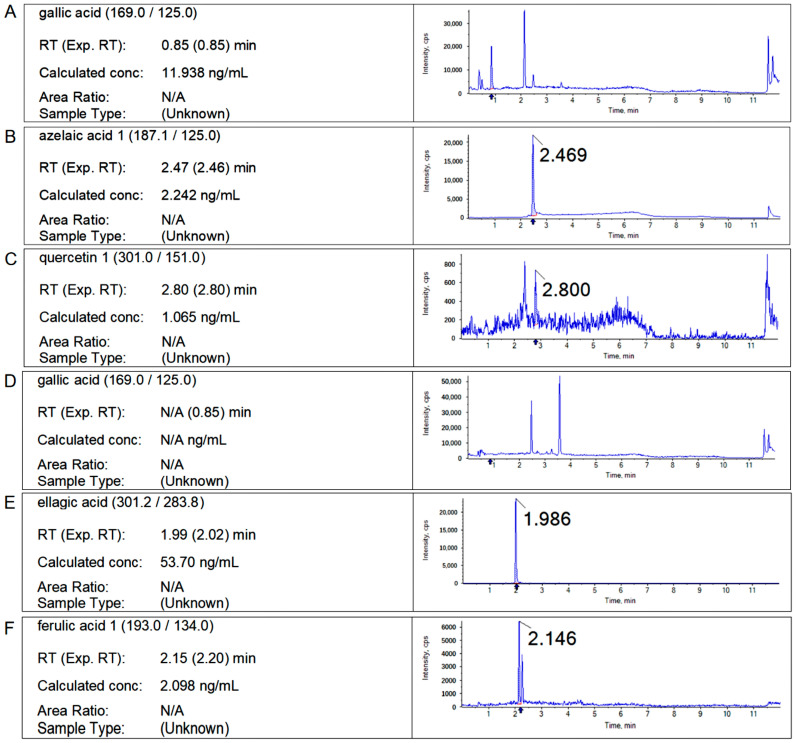
Chromatograms of free polyphenols and bound polyphenols in *Quercus dentata* kernel. (**A**–**C**): The chromatograms of free gallic acid, azelaic acid, and quercetin in the kernel extract of *Quercus dentata*. (**D**–**F**): The chromatograms of bound gallic acid, ellagic acid, and ferulic acid in the kernel extract of *Quercus dentata*.

According to the experimental method in Section 4.3, the retention time of the standard sample and the characteristic fragment ion information, and the contents of free polyphenols and bound polyphenols in *Q. variabilis*, *Q. aliena,* and *Q. dentata* acorns were determined, respectively, and the chromatograms of the samples are shown as follows (Figure 2, Figure 3 and Figure 4).

### 2.3. Content Analysis of Main Polyphenols

Based on the total polyphenol content (TPC) determination method described in Section 4.2, we measured the TPC content in *Q. variabilis*, *Q. aliena*, and *Q. dentata*, as shown in Table 1. Additionally, using the method described in Section 4.3, we accurately determined the content of each polyphenol in the acorns, as presented in Table 2. The results showed that the contents of free polyphenols in acorn kernels of *Q. variabilis*, from highest to lowest, included gallic acid, azelaic acid, and quercetin. The content of gallic acid was about 23.85 times that of quercetin and 2.59 times that of azelaic acid. In the free polyphenol extract of *Q. aliena* acorn kernels, the content of gallic acid is higher than those of quercetin and azelaic acid. The results of the quantitative analysis show that the content of gallic acid is about 9.86 times that of quercetin and 51.63 times that of azelaic acid. For the free polyphenol extract from acorn kernels of *Q. dentata*, the content of gallic acid is higher than those of azelaic acid and quercetin. The content of free gallic acid is about 11.21 times that of quercetin and 5.32 times that of azelaic acid. In bound polyphenol extract from *Q. variabilis* acorn kernels, the content of ellagic acid is higher than those of ferulic acid and gallic acid, among which the ellagic acid content is 30.46 times that of ferulic acid and 32.19 times that of bound gallic acid. In the free polyphenol extract of *Q. aliena* acorn kernels, the content of ellagic acid is higher than that of gallic acid, and that of gallic acid is higher than that of ferulic acid, among which the content of ellagic acid is 7.42 times that of combined gallic acid and 47.52 times that of ferulic acid. The content of ellagic acid in the combined polyphenol extract of *Q. dentata* is 25.59 times that of ferulic acid. However, the bound gallic acid was not detected.

### 2.4. Comparison of the Effects of Main Acorn Polyphenols on the Progress of the Disease Course in AD Mice

**Figure 5:**
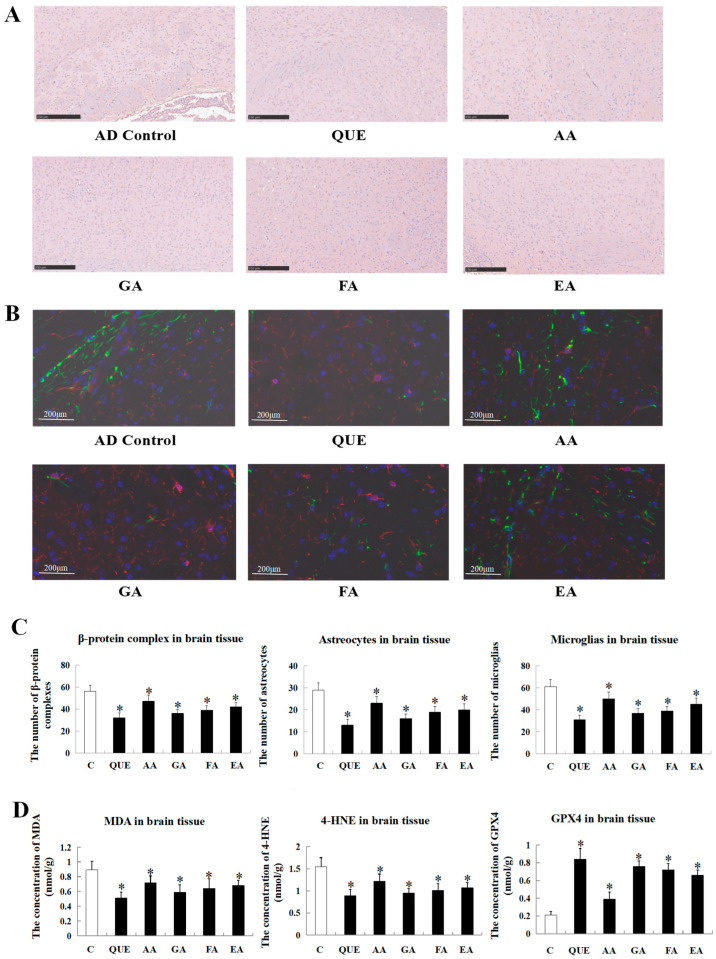
The alleviation of five main components of acorn polyphenols in the progression of AD. (**A**) IHC staining of Aβ protein in brain tissue; (**B**) Dual immunofluorescence staining of astrocytes/microglia (GFAP/Iba-1) in mouse brain tissue; (**C**) quantification of amyloid-beta protein, astrocytes, and microglia in brain tissue; (**D**) determination of physiological indicators in brain tissue (MDA, 4-HNE, GPX4). The mice in the experimental group were given 10 μM of the compound orally daily (total volume: 200 μL), namely gallic acid (0.085 g/kg b.w.), quercetin (0.15 g/kg b.w.), azelaic acid (0.090 g/kg b.w.), ferulic acid (0.092 g/kg b.w.), and ellagic acid (0.15 g/kg b.w.). * stood for the statistical difference when compared with the control group (**C**).

After the intervention of five compounds on APP/PS1 transgenic mice, the total number of Aβ precipitates in brain tissue decreased to different degrees (Figure 5). The area of Aβ precipitates in the brain tissue of mice in each group is shown in Figure 5A,C. The results show that the amounts of Aβ precipitates in each group from smallest to greatest are as follows: the QUE group, EA group, FA group, GA group, AA group, and model group. Compared with the model group, the precipitation of Aβ in the brain tissue of the QUE group was significantly reduced, which indicated that quercetin had the best down-regulation effect on the precipitation of Aβ in the brain tissue of AD mice. As shown in Figure 5B, compared with the model group, the signal intensity of GFAP and Iba-1 in the brain tissue of mice intervened on by five compounds decreased, indicating that all five compounds can reduce the expression levels of GFAP and Iba-1 protein. The data analysis is shown in Figure 5C. The numbers of microglia and astrocytes in the brain tissue of mice in each group are in the following order, from highest to lowest: the QUE group, EA group, FA group, GA group, AA group, and model group. The oxidative indicators MDA and 4-HNE were down-regulated by these chemicals to different degrees, and the level of the anti-oxidation indicator GPX4 was up-regulated with the treatment of these chemicals, which showed a similar tendency with the results in Figure 5A–C (Figure 5D). These results are consistent with the precipitation amount of Aβ, indicating that acorn polyphenols might be potential drugs for treating Alzheimer’s disease, and quercetin, the main component of acorn polyphenols, might have the best therapeutic effect on Alzheimer’s disease.

## 3. Discussion

Acorns are abundant sources of plant polyphenols due to their high concentration of phenolic compounds. However, there is a lack of qualitative and quantitative analysis regarding polyphenols in acorns of common oak species in China. Attentionally, the exploitation and utilization of acorn resources are insufficient, so it is urgent to develop them as homologous resources for medicine and food. Therefore, we investigated acorns from three prevalent tree species, namely *Q. variabilis*, *Q. aliena*, and *Q. dentata*, as raw materials.

Ultrasound-assisted extraction is an effective method for extracting plant polyphenols. Most of the related literature uses ultrasonic instruments for assisted extraction [3,18,19]. In the present study, an ultrasonic cell disintegrator was used to improve polyphenol extraction efficiency and yield more effectively. Optimal parameters for extracting acorn polyphenols by ultrasonic cell disruption were proposed—an ultrasonic power of 40%, an ultrasound on-time of 1 s, an ultrasound off-time of 2 s, and a total work time of 40 min—to achieve more efficient extraction.

The extraction of polyphenols traditionally targets only free polyphenols. In our research, after extracting the free polyphenols, alkaline hydrolysis was employed to liberate the bound polyphenols. This broadened the range of polyphenol extraction and improved the utilization of acorn resources. For the crude extraction purification of acorn polyphenols, HPD-600 and AB-8 macroporous resins were selected, and HPD-600 was found to be more suitable for purifying free polyphenols from acorns, while AB-8 could effectively separate bound polyphenols. Dynamic adsorption was used to purify acorn kernel polyphenols, improving purification efficiency, reducing operation time, and allowing for better control of adsorption and elution conditions, which obviously contributed to increased purity, reduced impurities, and more precise detection of the main polyphenol components. HPLC-MS was used to detect the main components in the extracts. The results indicated that acorn kernels from both *Q. variabilis* and *Q. aliena* could be used for ellagic acid extraction. *Q. aliena* acorn kernels were the most suitable for scientific research or production work related to the incorporation of gallic acid.

Alzheimer’s disease, the most common degenerative disease of the central nervous system, is characterized by significant memory loss and cognitive impairments, making it the most prevalent type of dementia among the elderly. Research indicates that acorn polyphenols have considerable potential in reducing Aβ deposition, alleviating oxidative stress, and modulating anti-inflammatory pathways [20,21], suggesting they could prevent or slow the onset of AD [22,23]. Our experiments corroborated these findings, showing reductions in Aβ deposition, oxidative stress markers (MDA and 4-HNE), and neuroinflammatory markers (GFAP and Iba-1) in AD mouse brain tissue treated with acorn polyphenols, highlighting their potential to delay AD progression.

Specifically, among the acorn polyphenols, five compounds were proved to inhibit the progression of AD in the following order—QUE > GA > FA > EA > AA—and they might take effect through different mechanisms. Multiple studies have shown that QUE and GA are widely considered potential therapeutic agents for alleviating, preventing, or slowing age-related neurodegenerative changes [24,25,26,27,28], which achieve the effects by inhibiting Aβ deposition and down-regulating inflammatory markers. Our experimental findings strongly aligned with the above report, as we observed that both QUE and GA significantly inhibited the progression of AD across various indicators. Additionally, FA was validated to reduce microglial aggregation, with our results indicating a significant decrease in GFAP and Iba-1 levels, suggesting polyphenolic compounds might mitigate the activation of astrocytes and microglia [29,30], but its role in AD has not been uncovered yet. Our experiments also demonstrated that AA alleviated AD injuries, which provides a new strategy for subsequent studies.

Additionally, research has shown that polyphenols, particularly EA, possess metal chelating properties, playing a significant role in mitigating metal-induced oxidative stress, such as ferroptosis [31]. The accumulation and aberrant metabolism of iron, leading to lipid peroxidation, are key conditions for ferroptosis [32,33]. Notably, our study found that under the influence of acorn polyphenol compounds like EA, there was a significant increase in GPX4 and a decrease in MDA and 4-HNE in AD mouse brain tissue. Considering GPX4 is an antioxidant enzyme utilizing GSH as a cofactor, it reduces the formation of lipid peroxides, showing the contrary effects with MDA/4-HNE. This discovery suggests that acorn polyphenol compounds, especially EA, might prevent neuronal ferroptosis by addressing iron accumulation and its abnormal metabolism, thereby effectively alleviating the progression of AD. To conclude, based on the special effect evaluation of acorn polyphenols, the present study offers a basis for research and development of new medicines; the gathered data also provide a new therapeutic approach for AD clinical treatment.

## 4. Materials and Methods

### 4.1. Materials and Reagents

**Figure 6:**
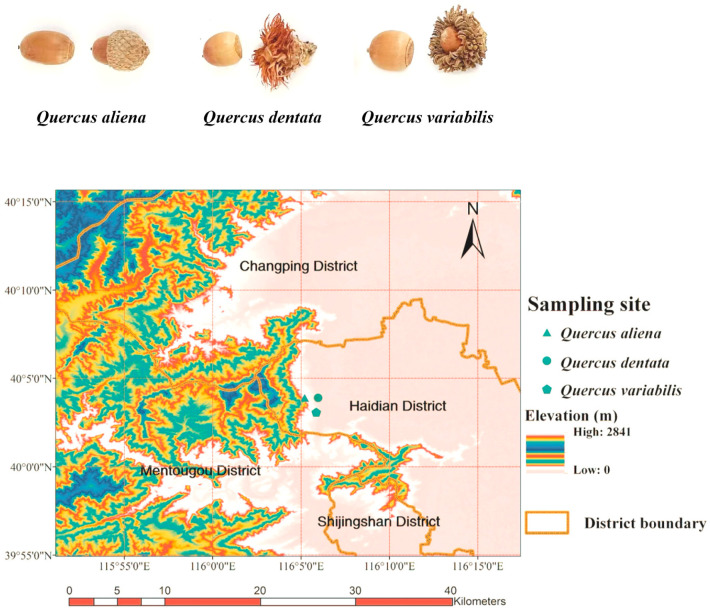
Acorn sample gathering information. Acorn fruits of three tree species, *Quercus variabilis*, *Quercus aliena*, and *Quercus dentata*, and geographic location of acorn samples in Jiufeng mountain, Beijing, China.

Three types of acorns (*Q. variabilis*, *Q. aliena*, and *Q. dentata*) were harvested from Jiufeng mountain in Beijing (the geographic location of the acorn samples in Jiufeng mountain is shown in Figure 6). The main reagents used were as follows: 30% ethanol, 70% ethanol, absolute ethanol, petroleum ether (30°~60°), ethyl acetate, distilled water, 5% sodium hydroxide solution, 2 M sodium hydroxide aqueous solution, 5% hydrochloric acid solution, concentrated hydrochloric acid (12.1 M), AB-8 macroporous resin, HPD-600 macroporous resin, 12% sodium carbonate solution, Folin-phenol reagent, acetonitrile (ACS grade), methanol (ACS grade), and formic acid (from China National Pharmaceutical Group Chemical Reagent Co., Ltd., Beijing, China).

APP/PS1 transgenic AD mice, aged 3 months, were acquired from Huachuangxinnuo Company (Nanjing, China). The associated antibodies for fluorescent double staining of GFAP/Iba-1 were obtained from Abcam Company (Shanghai, China). IHC staining kits (Aβ) were purchased from YaJi (Shanghai, China). Specialized ELISA kits (MDA, 4-HNE, and GPX4) and other reagents were obtained from Solarbio (Beijing, China).

### 4.2. Extraction and Purification of Free and Bound Polyphenols from Acorns

The fresh acorn samples were washed and then dried using hot air at 45 °C for 48 h. Once the acorn shells cracked, the kernels were separated from the shells. The kernels were then ground using a grinder and sieved through an 80-mesh screen for later use. The acorn powder was weighed at 25 g and 30% ethanol was added at a ratio of 1:30. Subsequently, the mixture was placed into an ultrasonic oscillation cell disruptor (Scientz, JY92-1IN, Ningbo, China). The ultrasonic power was set at 40%, with an on-time of 1 s and an off-time of 2 s, for a total working time of 40 min. After repeating the aforementioned steps three times, the extraction was completed. The extraction solution was centrifuged at 12,000× *g* for 15 min, resulting in the separation of supernatant and precipitate. The precipitate was retained and combined with the supernatant. After being washed three times with petroleum ether (twice the volume of the concentrated liquid) to remove fats, the mixture underwent vacuum rotary evaporation (40 °C) to remove organic solvents, followed by freeze-drying to obtain crude polyphenol samples. A wet-column chromatography method was utilized to pack the crude polyphenol samples, followed by dynamic adsorption using HPD-600 macroporous resin for purification of the polyphenols from acorn kernels. The eluent was concentrated under a vacuum and dried to obtain pure polyphenols from acorns.

The precipitated acorn powder after extraction of free polyphenols (25 g) was dispersed in a 500 mL solution of 2 M sodium hydroxide. The dispersion was then subjected to ultrasonic disruption using an ultrasonic cell disruptor. The ultrasonic power of the disruptor was set to 40%, with an on-time of 1 s and an off-time of 2 s, for a total working time of 40 min. After repeating this process three times, the extraction was completed. Subsequently, the solution was hydrolyzed at room temperature for 4 h. The pH of the solution was acidified to 2.0 with concentrated hydrochloric acid (12.1 M), followed by extraction with 3 × 500 mL of ethyl acetate. The solution was then concentrated to 1 mL under reduced pressure at 45 °C. The solution was purified by AB-8 macroporous resin column (the same as the purification procedure for free polyphenol extract), and then the purified combined polyphenol extract was obtained. The total polyphenol content was measured using the Folin–Ciocalteu method and reported as milligrams of gallic acid equivalents per gram of dry weight (mg GAE/g d.w.).

### 4.3. Analysis of Free or Bound Polyphenols by HPLC-MC/MS

An appropriate amount of the sample was added to 2 mL of 70% methanol and shaken for 1 min, followed by ultrasonic extraction for 30 min. After this, the supernatant was centrifuged and passed through a 0.22 μm filter membrane for detection. The column was Agilent C18 (2.1 mm ∗ 100 mm, 1.8 μm). The column temperature of the LC-M was set at 35 °C, the flow rate was 0.3 mL/min, and the acquisition mode was ESI-. The mobile phase was A: 0.1% formic acid in water and B: acetonitrile. Chromatographic conditions: 0–0.5 min, A: 90%, B: 10%; 0.5–4 min, A: 10%, B: 90%; 4–5.5 min, A: 90%, B: 10%. Mass spectrometry conditions: the scanning mode was used with the ion source temperature at 230 °C, quadrupole temperature at 150 °C, and transmission line temperature at 250 °C. The target content was calculated according to the following formula:

W = [(C − C0) * V * N]/*m* (1)

where

W—the content of the target substance in the sample, in mg/kg;

C—the concentration of the target substance in the sample determination solution, in mg/L;

C_0_—the concentration of the target substance in the blank control, in mg/L;

V—the constant volume, in mL;

N—the dilution multiple;

*m*—the sampling amount of the sample, in g.

### 4.4. Animal Model Construction

Thirty SPF male APP/PS1 transgenic mice (3 months old) were used as experimental subjects. These mice weigh about 20 ± 2 g. Mice were randomly divided into 6 groups (5 mice in each group), namely a model group without any treatment, the QUE treatment group, the AA treatment group, the GA treatment group, the FA treatment group, and the EA treatment group. The mice in the experimental group were given 10 μM of the compound orally daily (total volume: 200 μL), namely gallic acid (0.085 g/kg b.w.), quercetin (0.15 g/kg b.w.), azelaic acid (0.090 g/kg b.w.), ferulic acid (0.092 g/kg b.w.), and ellagic acid (0.15 g/kg b.w.). And the mice in the model group were given the same volume of sterile normal saline orally. All the mice were sacrificed after 5 weeks, and the blood samples and the organs were collected for the further detections. The animal experiments in this study were approved by the Ethics Committee of the Sinoresearch Biotechnology Co., Ltd. (Beijing, China; ZYZC20240201S).

### 4.5. Specific Marker Staining of the Brain Tissue (GFAP/Iba-1 Fluorescence Double Staining and Aβ)

Experimental mouse brain tissues were prepared as paraffin sections with a thickness of 3–4 μm for subsequent staining observations. Initially, IHC staining kits were utilized for the specific staining of Aβ to observe the progression of Alzheimer’s disease. A Nikon Eclipse 800 microscope (Nikon, Tokyo, Japan) was used to observe the tissue sections stained for IHC (Aβ). Subsequently, for GFAP/Iba-1 fluorescent double staining, the tissues were blocked with 5% NGS/2% BSA/1.5% Triton/PBS. Mouse brain tissue sections were incubated with the corresponding anti-GFAP and anti-Iba-1 antibodies (1:100) at 4 °C overnight. After the sections were washed with PBS to remove unbound primary antibodies, they were incubated with secondary antibodies labeled with different fluorescent dyes. The sections were washed again with PBS to remove any unbound secondary antibodies, preparing them for subsequent observation. GFAP/Iba-1 stained tissue sections were observed using a confocal laser scanning microscope (Olympus, Tokyo, Japan).

### 4.6. Detection of MDA, 4-HNE, and GPX4 in Brain Tissue of AD Mice

According to the special ELISA kits (MDA, 4-HNE and GPX4), the brain tissue was subjected to routine homogenate treatment and centrifuged at 4 °C and 12,000× *g* for 15 min, after which the supernatant was incubated with thiobarbituric acid at 95 °C for 75 min and placed on ice for 10 min. The absorbance of the supernatant was observed at a wavelength of 532 nm.

## 5. Conclusions

To conclude, in the present study, we primarily improved several technical details to broaden the range of polyphenol extraction and improve the utilization of acorn resources, including using alkaline hydrolysis, HPD-600 and AB-8 macroporous resins, a dynamic adsorption system, etc. Through comparing the analysis results, we found that acorn kernels from both *Q. variabilis* and *Q. aliena* could be used for ellagic acid extraction, and *Q. aliena* acorn kernels were the most suitable for scientific research or production work related to the incorporation of gallic acid. Then, in in vivo experiments, five main components of acorn polyphenols were proved to reduce Aβ deposition, alleviate oxidative stress, and modulate anti-neuroinflammatory markers, highlighting their potential to suppress the development of AD progression.

## Figures and Tables

**Table 1:** Total polyphenol content (TPC) in three different acorns.

Total Phenolic Content (TPC)	*Q. variabilis* (mg GAE/g d.w.)	*Q. aliena* (mg GAE/g d.w.)	*Q. dentata* (mg GAE/g d.w.)
Free polyphenol	1.32	1.07	1.21
Bound polyphenol	133.70	64.38	8.45

**Table 2:** Contents of main polyphenols in three different acorns.

Chemical Compound	Extract of* Quercus variabilis* Acorn Nutlet (mg/kg)	Extract of *Quercus aliena* Acorn Kernel (mg/kg)	Extract of *Quercus dentata* Acorn Kernel (mg/kg)
Free gallic acid	134.28	105.86	119.38
Quercetin	5.63	10.74	10.65
Azelaic acid	51.84	2.05	22.42
Bound gallic acid	2098.33	3971.75	0
Ferulic acid	2217.52	620.05	2.098
Ellagic acid	67,537.04	29,471.77	53.7

## Data Availability

The datasets used and/or analyzed in this study are available from the corresponding author upon reasonable request.

## Supplementary Materials

The following supporting information can be downloaded at: https://www.mdpi.com/article/10.3390/ijms251910536/s1.
