# Correction: Du et al. Extraction Condition Optimization, Quantitative Analysis, and Anti-AD Bioactivity Evaluation of Acorn Polyphenols from *Quercus variabilis*, *Quercus aliena*, and *Quercus dentata*. *Int. J. Mol. Sci.* 2024, *25*, 10536

**DOI:** 10.3390/ijms26052015

**Published:** 2025-02-26

**Authors:** Jianing Du, Zhengkun Han, Longyi Ran, Taiyu Zhang, Junru Li, Huiying Li

**Affiliations:** Beijing Key Laboratory of Food Processing and Safety in Forestry, College of Biological Sciences and Technology, Beijing Forestry University, Beijing 100083, China; djn15661258236@bjfu.edu.cn (J.D.); zhengkunh1005@126.com (Z.H.); rly2229@bjfu.edu.cn (L.R.); zhangtaiyu1120@163.com (T.Z.); 18395270921@163.com (J.L.)

In the original publication [1], there was an error in Section 4.4, where the ethical approval number was incorrectly stated as “ZYZC202402006S”. The correct ethical approval number should be “ZYZC20240201S”.

A correction has been made to Section 4.4, and the revised text should read: “The animal experiments in this study were approved by the Ethics Committee of the Sinoresearch Biotechnology Co., Ltd. (Beijing, China; ZYZC20240201S)”.

The authors apologize for this error and confirm that this correction does not affect the scientific conclusions of the paper. To ensure complete transparency and compliance with journal policies, all related materials, including supporting documentation, have been compiled and will be submitted alongside this correction request. The authors appreciate the journal’s consideration and cooperation in addressing this matter.
