# Supplementary material for: Extraction Condition Optimization, Quantitative Analysis, and Anti-AD Bioactivity Evaluation of Acorn Polyphenols from Quercus variabilis, Quercus aliena, and Quercus dentata
Source: Int J Mol Sci. 2024 Sep 30;25(19):10536. doi: 10.3390/ijms251910536 (PMC11476354; doi:10.3390/ijms251910536)
Supplement: Supplementary file 1 [file ijms-25-10536-s001.zip › ijms-3161367-supplementary.pdf]

Supplementary Table S1 Linear regression equations of major polyphenols

Chemical compound	Linear regression equation	R^2
Gallic acid	y=90.680568*x+6.441967	0.99985483
Ellagic acid	y=5.281332*x-23.678633	0.99933224
Ferulic acid	y=20.637412*x+3.438542	0.99931156
Azelaic acid	y=58.663119*x+273.104601	0.99968912
Quercetin	y=157.483081*x+38.409892	0.99936772

Supplementary Table S2 Retention times of major polyphenols

Standard sample	1.0000 (ng/mL)	5.0000 (ng/mL)	10.0000 (ng/mL)	50.0000 (ng/mL)	100.0000 (ng/mL)	500.0000 (ng/mL)	1000.0000 (ng/mL)	Mean value of retention time (min)
Gallic acid	0.673	0.697	0.697	0.697	0.697	/	/	0.692
Ellagic acid	/	/	1.860	1.868	1.852	1.860	1.876	1.863
Ferulic acid	1.949	1.965	1.957	1.957	1.949	1.957	1.973	1.958
Azelaic acid	2.037	2.045	2.045	2.045	2.037	2.045	/	2.042
Quercetin	2.215	2.223	2.223	2.223	2.215	/	/	2.220

Supplementary Table S3 Characteristic fragment ion mass-to-charge ratio of each reference sample

Standard sample	Mass-to-charge ratio
Gallic acid (GA)	169.0/125.0
	169.0/79.1
Ellagic acid (EA)	301.2/283.8
	301.2/144.9
Ferulic acid (FA)	193.0/178.0
	193.0/134.0
Azelaic acid (AA)	187.1/169.1
	187.1/125.0
Quercetin (QUE)	301.0→179.0
	301.0→151.0
